# Antimicrobial activity and mechanisms of *Salvia sclarea* essential oil

**DOI:** 10.1186/s40529-015-0096-4

**Published:** 2015-06-19

**Authors:** Haiying Cui, Xuejing Zhang, Hui Zhou, Chengting Zhao, Lin Lin

**Affiliations:** School of Food & Biological Engineering, Jiangsu University, Zhenjiang, 212013 P. R. China

**Keywords:** *Salvia sclarea*, Essential oil, Antibacterial activity, Antibacterial mechanisms

## Abstract

**Background:**

Nowadays, essential oils are recognized as safe substances and can be used as antibacterial additives. *Salvia sclarea* is one of the most important aromatic plants cultivated world-wide as a source of essential oils. In addition to being flavoring foods, *Salvia sclarea* essential oil can also act as antimicrobials and preservatives against food spoilage. Understanding more about the antibacterial performance and possible mechanism of *Salvia sclarea* essential oil will be helpful for its application in the future. But so far few related researches have been reported.

**Results:**

In our study, *Salvia sclarea* oil showed obvious antibacterial activity against all tested bacterial strains. Minimum inhibitory concentration (MIC) and minimum bactericide concentration (MBC) of seven pathogens were 0.05 and 0.1 % respectively. In addition, *Salvia sclarea* oil also exhibited a significant inhibitory effect on the growth of *Escherichia coli* (*E. coli*) in phosphate buffer saline (PBS) and meats. After treated with *Salvia sclarea* oil, Scanning Electron Microscope (SEM) images can clearly see the damage of cell membrane; the intracellular ATP concentrations of *E. coli* and *S. aureus* reduced 98.27 and 69.61 % respectively, compared to the control groups; the nuclear DNA content of *E. coli* and *S. aureus* was significantly reduced to 48.32 and 50.77 % respectively. In addition, there was massive leakage of cellular material when *E. coli* and *S.* aureus were exposed to *Salvia sclarea* oil.

**Conclusions:**

*Salvia sclarea* essential oil damaged the cell membrane and changed the cell membrane permeability, leading to the release of some cytoplasm such as macromolecular substances, ATP and DNA. In general, the antimicrobial action of *Salvia sclarea* essential oil is not only attributable to a unique pathway, but also involves a series of events both on the cell surface and within the cytoplasm. Therefore, more experiments need to be done to fully understand the antimicrobial mechanism of *Salvia sclarea* essential oil.

## Background

The genus *Salvia* is one of the largest and the most important aromatic and medicinal genera of the *Lamiaceae* family and comprises about 900 species, widespread throughout the world (Russo et al. 2013). Since ancient times, *Salvia sclarea* is one of the most appreciated medicinal herbs native to Mediterranean countries, and widely used in medicine and cooking (Durling et al. 2007), as well as in cosmetics, perfumery and the pharmaceutical industry (Kong et al. 2010). Plants from this genus are renowned for their biological activities such as antibacterial, antioxidant, antitumor, antidiabetic, antimicrobial, anxiolytic, sedative and anti-inflammatory activities (Rajabia et al. 2014).

In the world, the serious consequences caused by food-borne disease every year make food safety issues become the focus of attention by people and the priority issues of global public health (Cao et al. 2013). As a kind of the necessary food in our life, meat and its products are rich in protein, lipids and have suitable moisture content, which makes them to be the ‘natural media’ of microorganisms (Álvarez-Fernández et al. 2013), and they can be easily contaminated by *E. coli* during animal evisceration after slaughter, through contact with tainted water or during meat handling (Newell et al. 2010). So in spite of modern improvements in slaughter hygiene and food production techniques, the food safety of meat products is still an increasingly important public health issue (Burt 2004). To minimize meats degradation by microorganisms and extend the shelf life of meats, legislation allows the application of antibacterial additives (Guillard et al. 2009). However, excessive use of antibacterial additives encourages the emergence of resistant bacteria. Bacteria contaminated nearly half of U.S. meat samples in a recent analysis, with 96 % of bacteria showing resistance to at least one type of drug (Fadli et al. 2012). So, due to the potential risk with synthetic antibacterial agents, there is a growing interest in the use of natural antimicrobials, particularly those that are derived from plant sources, for example, essential oils.

Essential oils (EOs) are aromatic oily liquids obtained from various plants (Marianne 2011). They are a group of secondary metabolites, complex mixtures of terpenoids, with characteristic flavor and fragrance properties (Stefanakis et al. 2013). So far, EOs play an important role in the protection of food safety as antibacterials, antivirals, antifungals, and antioxidants (Pandey et al. 2014). Nowadays essential oils are recognized as safe substances by the Food and Drug Administration and some contain compounds which can be used as antibacterial additives (Tian et al. 2014). A large number of reports concerning the antioxidant and the antimicrobial ability of essential oils have already been published. EOs become increasingly popular as natural antimicrobial and antioxidant agents that may be used in food preservation (Hossain et al. 2014; Jallali et al. 2014). Among many EOs, *Salvia sclarea* is an aromatic herb and thus was considered mainly for its essential oil content (Taarit et al. 2009; Alves-Silva et al. 2013). The essential oil is the main bioactive ingredient of *Salvia sclarea*. In addition to being flavouring foods, *Salvia sclarea* essential oil can also act as antimicrobials and preservatives against food spoilage (Kozics et al. 2013).

Understanding more about the antibacterial performance and possible mechanism of *Salvia sclarea* essential oil in meats will be helpful for its application in the future. But so far few related researches have been reported. Therefore, the aims of this study were a) to evaluate the antibacterial activity of the *Salvia sclarea* essential oil, and b) investigate the antibacterial mechanisms of *Salvia sclarea* essential oil.

## Methods

### Bacterial strains

The following bacterial strains were selected: *Escherichia coli* ATCC 25922, *Staphyloccocus aureus* ATCC 25923, *Bacillus pumilus* ATCC 27142, *Klebsiella pneumonia* ATCC 13883, *Bacillus subtilis* IFO 3457, *Salmonella typhi*murium B11, *Pseudomonas aeruginosa* ATCC 27853. The strains were cultured in Nutrient Broth (NB) at 35 °C for 24 or 48 h, and stored at −80 °C in NB.

### *Salvia sclarea* essential oil

The *Salvia sclarea* essential oil tested in this study was supplied by J.E International (French). linalyl acetate (74.562 %), linalol (12.326 %) and germacrene D (1.931 %) are the main constituents.

### Evaluation of antimicrobial activity

#### The antimicrobial activity of *Salvia sclarea* oil

*Salvia sclarea* essential oil was added into tubes containing NB to obtain concentrations of 0.1, 0.05, 0.025, 0.0125 and 0.00625 % respectively. Subsequently, the tubes were inoculated with the freshly prepared bacterial suspension in order to maintain initial bacterial concentration 10^3^–10^4^ CFU/mL, and then incubated in a rotary shaker at 150 rpm and 37 °C. The final treatment time of *Escherichia coli, Bacillus subtilis, Pseudomonas Aeruginosa, Bacillus pumilus* and *Salmonella typhimurium* is 24 h*; Staphylococcus aureus* and *Klebsiella pneumonia* is 48 h*.* The lowest concentration of essential oil showing growth inhibition (as seen visually) was considered as the minimum inhibitory concentration. The MBC was recorded as the lowest concentration of essential oil that showed no growth on Nutrient Agar (NA) plates after spot inoculation and incubation at 35 °C. The incubation time of *Escherichia coli, Bacillus subtilis, Pseudomonas Aeruginosa, Bacillus pumilus* and *Salmonella typhimurium* is 24 h; *Staphylococcus aureus* and *Klebsiella pneumonia* is 48 h (Bolou et al. 2011; Ruparelia et al. 2008). Each assay in this experiment was repeated three times.

### Kill time analysis of *Salvia sclarea* oil

The plate colony-counting method was used to analyze the kill time of *Salvia sclarea* oil. *Salvia sclarea* oil was diluted into tubes with PBS to obtain concentration of 0.1 %. Subsequently, the tubes were inoculated with the freshly prepared bacterial suspension in order to maintain initial bacterial concentration 10^5^ CFU/mL and cultured at 150 rpm and 37 °C. Numbers of viable bacteria were enumerated at 0, 0.5, 1, 2, 4 and 8 h by counting the number of bacterial colonies grown on the plate. As a control, the bacterial suspension in sterile PBS without *Salvia sclarea* oil was also tested (Petersen et al. 2006; Nakayama et al. 2012).

### Antimicrobial activity testing in meats

Chicken, pork and beef were bought from the local supermarket. The aseptic sample with an amount of 10 and 90 g water was blended into slurry. Subsequently, mixture was inoculated with 1 % (v/w) of *E. coli* suspension (1 × 10^4^ CFU/mL) and put into a sterilized 100 ml glass bottle. The samples with (0.1 v/v) or without *Salvia sclarea* oil were incubated at 30 °C for 24 and 48 h. Finally, the plate count method was carried out to determine the number of viable cells in the agar plate (Liu and Yang 2012).

### The bactericidal mechanism of *Salvia sclarea* oil

#### The integrity of the cell membrane

The size and morphology of the bacteria, *E. coli* and *S. aureus*, were examined by Scanning electron microscopy (SEM, JSM-7001F, JEOL, Japan). Firstly, *Salvia sclarea* oil was diluted into tubes with PBS to obtain concentration of 0.05 %, and then added to two test tubes that containing *E. coli* and *S. aureus*. The tubes were cultured at 150 rpm and 37 °C for 24 h. Then the bacteria cultures were fixed with 2.5 % glutaraldehyde for at least 2 h. The samples washed twice with PBS each for 20 min. The washed samples were put into 1 % osmium tetroxide for 4 h, followed by washing twice with PBS each 20 min. The samples were then dehydrated using sequential exposure per ethanol concentrations ranging from 50 to 100 % and washed twice with PBS each 20 min. The washed sample was then put onto a stub for air drying, coating with gold followed by microscopic examinations (Zhang et al. 2007).

### Loss of 260 nm-absorbing material

The release of cytosolic material absorbing at 260 nm from E. coli and S .aureus treated with *Salvia sclarea* oil at different concentrations, 0.25 MIC, 0.5 MIC, 1 MIC and 2 MIC, were performed on the bacteria suspension (10^8^ CFU/mL in PBS). After treatment at 37 °C for 20 h, cells were centrifuged at 4000 rpm for 15 min at 4 °C and the supernatant were filtered by microporous membrane filter. The absorbency of the filtrate at 260 nm was detected by ultraviolet spectrophotometer (UV-1801, Beijing, China). As a control group, a bacterial suspension in sterile PBS without *Salvia sclarea* oil was tested as well (Chami et al. 2005; Sharma et al. 2013).

### Measurement of cellular ATP concentrations

The broth of *E. coli* and *S. aureus* (10^8^ CFU/mL) were centrifuged for 10 min at 8000 rpm. The cell pellets were washed three times and resuspended in the buffer to make up 10^8^ CFU/mL suspensions of bacteria. After 0.05 % *Salvia sclarea* oil was added, the samples were cultured at 150 rpm and 37 °C for 30 min. Then, the samples were centrifuged for 10 min at 8000 rpm, and the cell pellets were collected. Finally, the cellular ATP concentrations of samples were determined by the Clean Sense TM Surface Hygiene Test Kit (LEYU Biotechnology, Shanghai, China). As a control group, the samples without *Salvia sclarea* oil were tested (Finger et al. 2012).

### Observed nucleic acids with fluorescent staining method

*S. aureus* was cultured at 37 °C with gentle agitation for 48 h, and *E. coli* for 24 h. Subsequently, the cells were collected and resuspended in PBS, then added in 0.05 % (v/v) *Salvia sclarea* oil and cultured at 37 °C for 24 h. The nucleic acids were observed using the diluted 4′6-diamidino-2- phenylindole (DAPI) staining method. Ten microliter of the sample solution treated with *Salvia sclarea* oil was dropped on a glass slide. Three times volume of diluted DAPI (10 μg/mL, Roche Diagnostics GmbH, Germany) was added to the glass slide and kept in the dark for 10 min. The nucleic acids were observed with fluorescent microscope (Leica DMI4000B). As a control group, the bacteria without *Salvia sclarea* oil were also detected (Wang et al. 2010).

### Measurement of the quantification of DNA

Centrifuge the suspensions at 4000 rpm for 15 min at 4 °C and the pellets were harvested. The pellets were washed three times with PBS and resuspended in the buffer containing 0.05 % (v/v) *Salvia sclarea* oil to make up bacterial suspensions of 10^8^ CFU/mL. Eight-hundred micro liters were removed from the suspensions at 0 h hand 24 h and immediately added to three times the volume of diluted DAPI and kept in the dark for 10 min. The fluorescence intensity of DNA was estimated using fluorescence spectrophotometer (Cary Eclpise, VARIAN, America) with the excitation wavelength of 364 nm. As control, the bacterial suspension in sterile PBS without *Salvia sclarea* oil was tested (Wang et al. 2010).

## Results and discussion

### Evaluation of antimicrobial activity

#### Determination of Minimum Inhibitory Concentration (MIC) and Minimum Bactericidal Concentration (MBC)

Obtained MIC and MBC values of the investigated isolates were summarized in Table 1. As can be seen, *Salvia sclarea* oil showed the strongest antibacterial activity against the investigated bacteria. Meanwhile *Salvia sclarea* oil showed equal bacteriostatic and bactericidal activity against gram-positive bacteria (*Staphylococcus aureus* and *Bacillus subtilis*) and gram-negative bacteria (*Escherichia coli*, *Salmonella typhimurium*, *Klebsiella pneumonia, Pseudomonas Aeruginosa, Bacillus pumilus*). The MICs of *Salvia sclarea* oil were both 0.05 %, and the MBCs were both 0.1 %. Those all indicated that *Salvia sclarea* oil was an effective bacterial inhibitor and bactericide with a broad antibacterial spectrum. Sepahvand et al. (2014) has demonstrated that *S. sclarea* essential oil did have different anti-bacterial effects with the magnitude *S. aureus = K. pneumonia* > *P. aeruginosa*. There are several reasons for the discrepancies of my observation and the previous report: (1) the difference among different species was obvious; (2) the antibacterial activities of *S. sclarea* essential oil were variable with different material and extraction methods; (3) there are differences between strains of the same origin. The difference between the strains of the same origin is resulted from long adaption to ecological environment, artificial selection and cross breeding. So the drug resistances of different strains can’t be exactly the same; (4) different experiment methods may lead to different results.

**Table 1:** MICs and MBCs of *Salvia sclarea* oil against 7 pathogens

Test strain	*Salvia sclarea* oil	
	MIC (%)	MBC (%)
*Escherichia coli*	0.05	0.1
*Staphylococcus aureus*	0.05	0.1
*Bacillus subtilis*	0.05	0.1
*Salmonella typhimurium*	0.05	0.1
*Klebsiella pneumonia*	0.05	0.1
*Pseudomonas Aeruginosa*	0.05	0.1
*Bacillus pumilus*	0.05	0.1

### The antimicrobial activity of *Salvia sclarea* oil testing in agar media

**Fig. 1:**
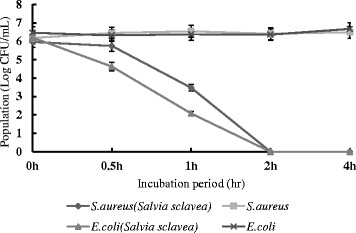
The antimicrobial activity of *Salvia sclarea* oil of *E. coli* and *S. aureus*

Due to equal bacteriostatic and bactericidal activity of *Salvia sclarea* oil against gram-negative bacteria and gram-positive bacteria, as the most representative gram-negative and gram-positive bacterium, *E. coli* and *S. aureus* were selected to further study to discuss the antibacterial activity and bactericidal mechanism of *Salvia sclarea* oil. From the Fig. 1, *Salvia sclarea* oil showed a good antimicrobial effect against both *E. coli* and *S. aureus*. About 99.99 and 99.9 % reduction in population were observed in *E. coli* and *S. aureus* respectively after 1 h of *Salvia sclarea* treatment. And after 2 h treatment, almost 99.9999 % reductions in population were achieved both in *E. coli* and *S. aureus*. In summary, *Salvia sclarea* oil could act as promising antimicrobials because of its short antimicrobial time.

### The antimicrobial activity of *Salvia sclarea* oil testing in meats

**Fig. 2:**
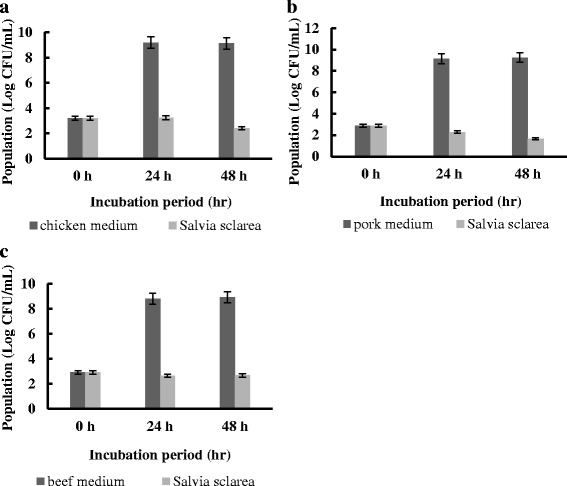
**a** The antibacterial activity of *Salvia sclarea* oil of *E. coli* on chicken medium (**b**) The antibacterial activity of *Salvia sclarea* oil of *E. coli* on pork medium (**c**) The antibacterial activity of *Salvia sclarea* oil of *E. coli* on beef medium

*Escherichia coli* (*E. coli*) are common in the normal microflora of the intestinal tract of humans and animals and are often used as an indicator of faecal contamination. Hence, the antibacterial activity of *Salvia sclarea* oil against *E. coli* on meat was investigated. As shown in Fig. 2, *Salvia sclarea* oil exhibited great antimicrobial activity on meat. After 48 h, almost 99.99999, 99.99999 and 99.9999 % reduction in population was observed on chicken medium, pork medium and beef medium respectively after treated by *Salvia sclarea* oil. The results indicated that *Salvia sclarea* oil had a marked effect on the inhibition of *E. coli* on meat. Therefore, *Salvia sclarea* oil can be regarded as a natural and efficient antiseptic of the pathogens which used on meat.

### The bactericidal mechanism of *Salvia sclarea* oil

#### Scanning Electron Microscope (SEM)

**Fig. 3:**
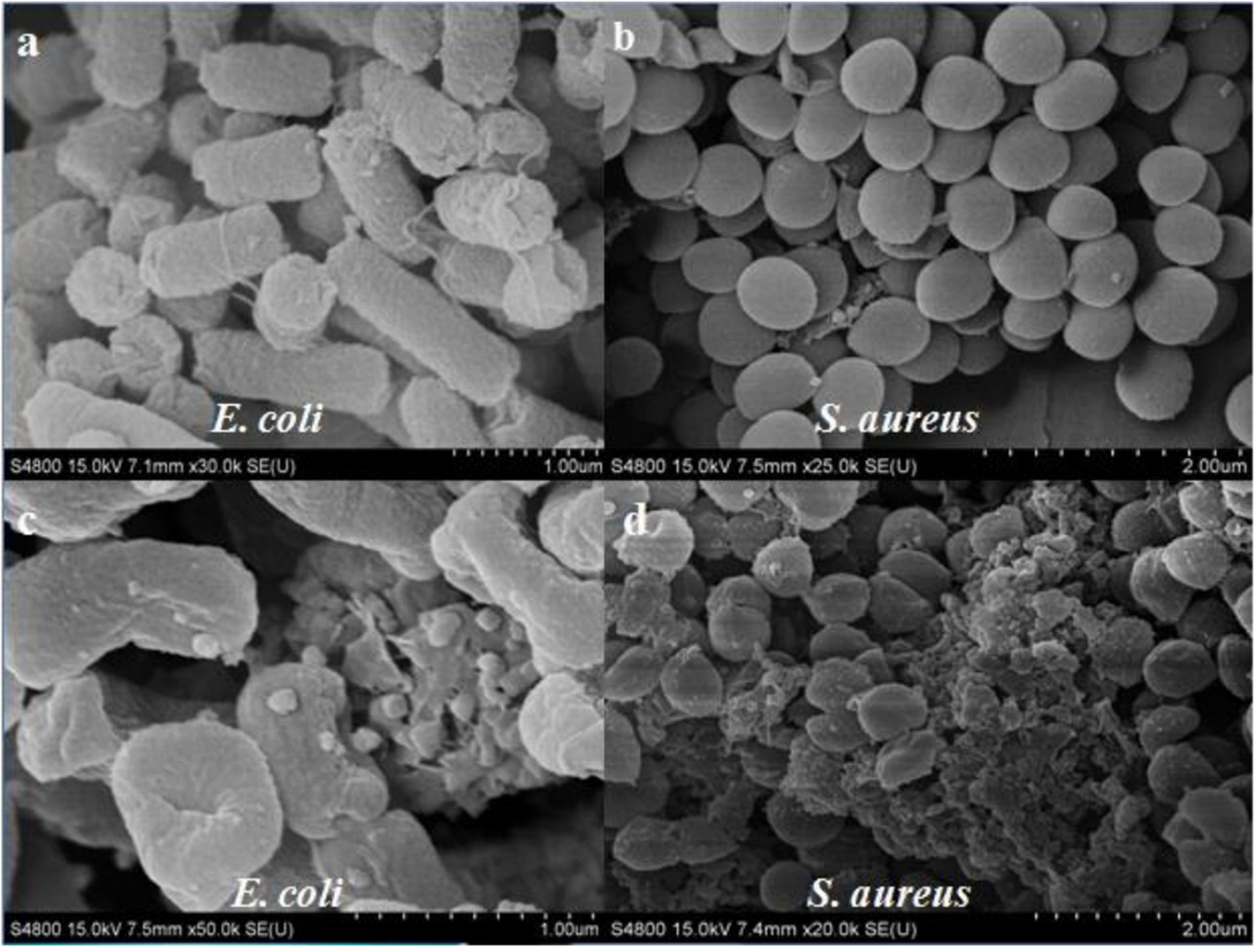
SEM images of *E. coli* and *S. aureus* before (**a**, **b**) and after (**c**, **d**) *Salvia sclarea* oil treatment. *Scale bar*: *E. coli* (1 μm); *S. aureus* (2 μm)

**Fig. 4:**
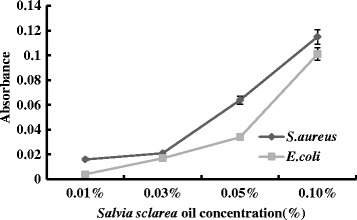
Changes in optical density at 260 nm of supernatant from *E. coli* and *S. aureus* cell suspension treated with *Salvia sclarea* oil

The surface morphology of *E. coli* and *S. aureus* cells were evaluated by SEM analysis. The electron micrographs of both control and *Salvia sclarea* oil treated microbial cells were presented in Fig. 4. In control groups, the untreated cells had the typical structure, showing a striated wall for *E. coli* (Fig. 3a) and a smooth wall for *S. aureus* (Fig. 3b). In contrast, obvious harmful effects on the morphology of cell membranes were presented when strains were treated with *Salvia sclarea* oil. Deformed, incomplete and pitted shapes were observed in treated *E. coli* and *S. aureus* (Fig. 3c and d).

### Loss of 260 nm-absorbing material

Changes in membrane integrity by *Salvia sclarea* oil cause the release of intracellular components. Small ions such as potassium and phosphate tend to flow out first, followed by macromolecular substances such as DNA and others (Lee et al. 2014). As we can see from Fig. 4, there was massive leakage of cellular material when *E. coli* and *S.* aureus were exposed to *Salvia sclarea* oil. Higher concentration of *Salvia sclarea* oil resulted in higher cell leakage, associated with loss of macromolecular substances. The level of cellular material reached a maximum after treatment with 0.1 % *Salvia sclarea* oil. And the results indicated that at higher concentration, the antibacterial activity of *Salvia sclarea* oil might be due to bactericidal damage to the membrane.

### Measurement of cellular ATP concentrations

**Fig. 5:**
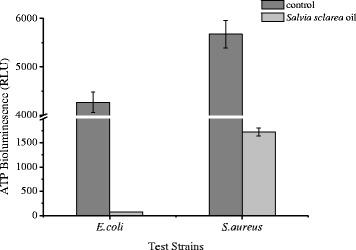
The cellular ATP concentrations of *E. coli* and *S. aureus* before and after *Salvia sclarea* oil treatment

To observe the level of *E. coli* and *S. aureus* membrane damage caused by *Salvia sclarea* oil, the amount of cellular ATP was measured by ATP bioluminescence assay (Fig. 5). As is shown above, cellular ATP concentrations of *E. coli* and *S. aureus* reduced after treated with *Salvia sclarea* oil. Results of ATP bioluminescence assay indicated the intracellular ATP concentrations of *E. coli* and *S. aureus* reduced 98.27 and 69.61 % respectively, compared to the control groups. The results suggest that *Salvia sclarea* oil affects membrane permeability and results in the release of ATP. Moreover, it is possible that *Salvia sclarea* oil acts against different bacterial enzymes, such as ATPase (Nazzaro et al. 2013). And the results also showed that the gram negative strains of bacteria were more sensitive than gram positive strains to *Salvia sclarea* oil.

### Nucleic acid staining

**Fig. 6:**
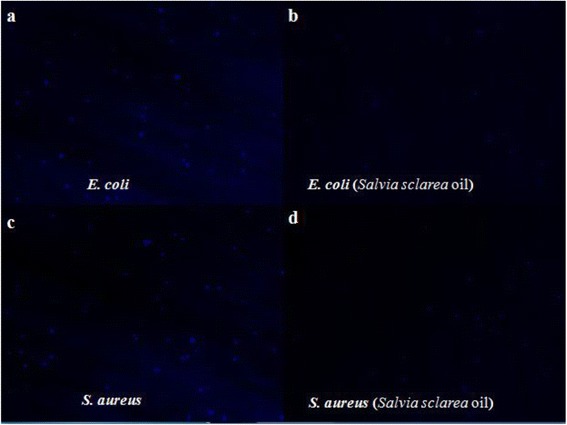
DNA fluorescence images of *E. coli* and *S. aureus* cells before (**a, c**) and after (**b, d**) *Salvia sclarea* oil treatment

**Fig. 7:**
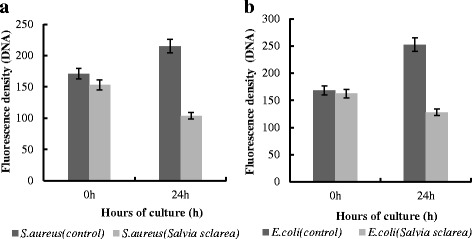
**a** The fluorescence density of DNA of *S. aureus*. **b** The fluorescence density of DNA of *E. coli*

DAPI is a fluorescent dye that could penetrate into the bacteria cells and integrate with DNA. The Fig. 6 showed that the fluorescence intensities of *E. coli* and *S. aureus* treated with *Salvia sclarea* oil (Fig. 6b and d) were obviously lower than control group. Similarly, the fluorescence spectrophotometer measurements indicated that the DNA content in *S. aureus* was significantly reduced to 48.32 % compared to the control group (Fig. 7a), and the DNA content in *E. coli* was reduced to 50.77 % (Fig. 7b). The results are the same as the fluorescent microscopic figures of *E. coli* and *S. aureus* cells treated with *Salvia sclarea* oil in Fig. 6. This implied that *Salvia sclarea* oil led to the decrease of DNA. Based on the results of the SEM images, this may be due to the interaction between *Salvia sclarea* oil and the bacterial cell membrane.

## Conclusion

The results of the study revealed that *Salvia sclarea* essential oil exhibited significant antibacterial activity against all seven tested bacterial strains in vitro. Moreover, the similar antibacterial performances were observed in different meats. It indicated that *Salvia sclarea* essential oil has strong broad-spectrum antimicrobial activity. *Salvia sclarea* essential oil damaged the cell membrane and changed the cell membrane permeability, leading to the release of the material inside the cell such as macromolecular substances, ATP and DNA. In general, the antimicrobial action of *Salvia sclarea* essential oil is not only attributable to a unique pathway, but also involves a series of events both on the cell surface and within the cytoplasm. Therefore, more experiments need to be done to fully understand the antimicrobial mechanism of *Salvia sclarea* essential oil. In addition, these studies provided an experimental basis of practical application of *Salvia sclarea* essential oil as a natural antibacterial agent.

## Abbreviations

MIC: Minimum inhibitory concentration
MBC: Minimum bactericide concentration
PBS: Phosphate buffer saline
NA: Nutrient Agar
SEM: Scanning electron microscope
DAPI: Diluted 4′6-diamidino-2- phenylindole
